# SMARCD3 is a potential prognostic marker and therapeutic target in CAFs

**DOI:** 10.18632/aging.104102

**Published:** 2020-10-28

**Authors:** Ming Jiang, Huiju Wang, Hong Chen, Yong Han

**Affiliations:** 1 Department of General Surgery, People’s Hospital of Quzhou, Quzhou, Zhejiang, China; 2 Clinical Research Institute, Zhejiang Provincial People’s Hospital, Zhejiang, China. People’s Hospital of Hangzhou Medical College, Zhejiang, China. Key Laboratory of Tumor Molecular Diagnosis and Individualized Medicine of Zhejiang Province, Zhejiang, China; 3 Department of Stomatology, Zhejiang Provincial People's Hospital, Hangzhou, China

**Keywords:** SMARCD3, colorectal cancer, tumor microenvironment, prognostic marker, therapeutic target

## Abstract

Objective: Screening for novel prognostic biomarkers and potential therapeutic targets from colorectal cancer microenvironment.

Results: 372 genes were overexpressed in colorectal cancer microenvironment, five of which that had the most prognostic powers were enriched in Epithelial-Mesenchymal Transition and cell cycle pathways. For the first time, we showed that SMARCD3 was mainly expressed in CAFs and could be a novel prognostic marker and potential therapeutic target. Function analyses indicated that MSARCD3 might promote CAFs activation and colorectal cancer metastasis through SMARCD3-WNT5A/TGF-β-MAPK14-SMARCD3 positive feedback loop. Signaling map of SMARCD3 was constructed and several potential drugs that could regulate SMARCD3 were also presented.

Conclusions: SMARCD3 is a novel prognostic biomarker and potential therapeutic target of colorectal cancer, which may promote cancer metastasis through activation of CAFs.

Methods: Colorectal cancer microenvironment related genes were screened based on immune and stromal scores. Function enrichment analyses were performed to show the underlying mechanistic insights of these tumor microenvironment related genes. Kaplan-Meier survival analysis was used for evaluating the prognostic power. Gene-Pathway interaction network analysis and cellular heterogeneity analysis of tumor microenvironment were also performed. Gene set enrichment analysis was performed for signal gene pathway analysis. Protein data from The Cancer Genome Atlas were used for validation.

## INTRODUCTION

Colorectal cancer is one of the most common digestive malignancies worldwide [1]. The tumor microenvironment (TME) plays critical roles in tumorigenesis, development, metastasis and therapeutic responses [2, 3]. For instance, PPM1H (Protein Phosphatase 1H) can inhibit the activation of SMAD signaling pathway and promote mesenchymal differentiation [4]. Knockdown the expression of PPM1H in pancreatic cancer cells can lead to increased expression of vimentin and changes of other epithelial or mesenchymal markers [4–6]. Recently, it is reported that PPM1H knockdown in colorectal cancer cells can induce vimentin expression and activate cancer-associated fibroblasts (CAFs), which in turn can promote the proliferation and migration of colorectal cancer cell with low PPM1H expression [7]. Overexpression and autocrine of WNT2 (Wnt Family Member 2) in CAFs can promote colon cancer proliferation, invasion and metastasis in vitro and in vivo [8, 9]. Exosomes released by CAFs can promote colorectal cancer metastasis and therapeutic resistance by inducing Epithelial-mesenchymal transition (EMT) and tumor cell stemness [10].

Nevertheless, the molecular mechanisms underlying TME associated colorectal cancer progression have never been well elucidated. Hence, screening for novel prognostic biomarkers and potential therapeutic targets for colorectal cancer from TME is of crucial importance. This study takes advantage of publicly available datasets and powerful bioinformatics tools to screen for genes with significant prognostic value and explore potential mechanistic insights.

## RESULTS

### Screening and function annotation of TME related DEGs in colorectal cancer

**Figure 1:**
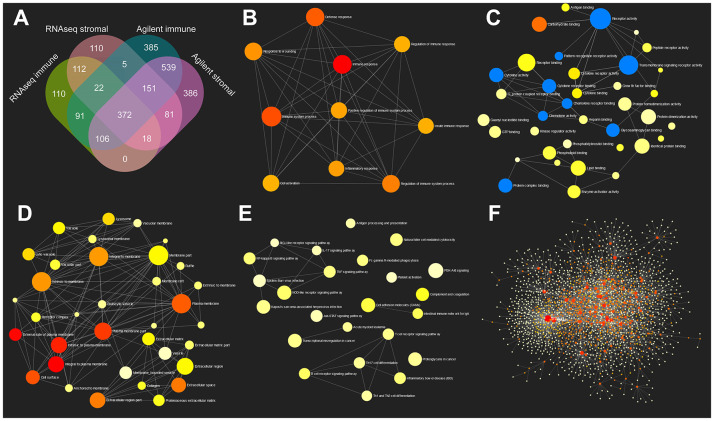
**GO and KEGG pathway enrichment network analysis of TME related genes.** (**A**) Venn diagram analysis of DEGs based on immune or stromal scores. (**B**–**E**) GO (biological process, molecular function and cellular component) and KEGG pathway enrichment network analysis of 372 commonly upregulated genes in TME. (**F**) Protein-protein interaction network of 372 TME related genes.

Immune score and stromal score of each sample were computed using ESTIMATE algorithm. TME related DEGs were computed based on immune and stromal scores using Agilent microarray expression data and RNAseq data of colon cancer from TCGA. The heat maps of DEGs based on different grouping strategies were presented in Supplementary Figure 1. Supplementary Figure 1A, 1B showed immune and stromal related DEGs computed using Agilent microarray data, while Supplementary Figure 1C, 1D showed DEGs computed using RNAseq data, respectively. Venn diagram analysis indicated that 372 genes were commonly upregulated in immune high and stromal high groups using either Agilent data or RNAseq data (Figure 1A). Details of these 372 genes were presented in Supplementary Table 1.

Gene ontology (GO: biological process, molecular function and cellular component) and KEGG analyses of these 372 genes were performed using network analyst (Figure 1B–1E). Protein-protein interaction (PPI) network was also constructed using network analyst (Figure 1F). As is shown in these figures, immune response, cytokine receptor activity and Toll-like receptor signaling pathway were significantly enriched. Detailed GO, KEGG and PPI results were shown in Supplementary Tables 2–6.

### Five genes were associated with poor survival and EMT

**Figure 2:**
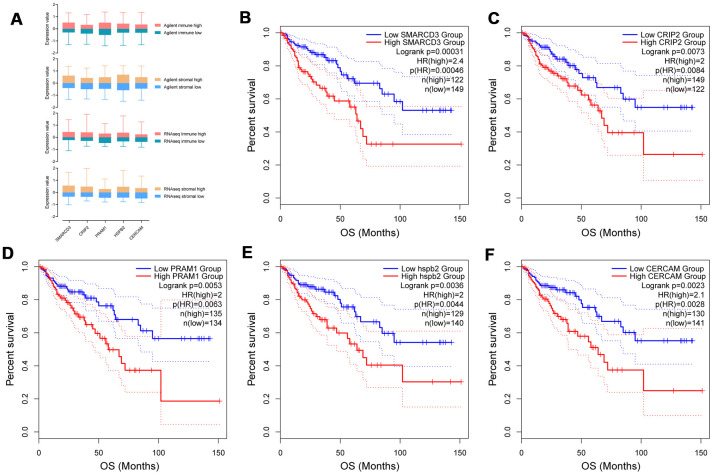
Expression of SMARCD3, CRIP2, PRAM1, HSPB2 and CERCAM in groups with different immune or stromal scores (**A**). Kaplan-Meier survival analysis based on expression value of these genes using TCGA COAD data (**B**–**F**).

Prognostic power of 372 commonly upregulated genes in TME were evaluated through Kaplan-Meier survival analysis using colorectal cancer data from TCGA. Five genes with most significant prognostic power were selected for further analyses. The expression levels of SMARCD3, CRIP2, PRAM1, HSPB2 and CERCAM in Agilent stromal/immune high/low groups and RNAseq stromal/immune high/low groups were demonstrated in Figure 2A. These five genes were all associated with poor OS of patients with colorectal cancer (Figure 2B–2F). Specifically, colorectal cancer patients with SMARCD3 high expression had poorer OS in comparison with patients with SMARCD3 low expression (Hazard ratio: 2.4, logrank p = 0.00031). Similarly, colorectal cancer patients with high expression of CRIP2, PRAM1, HSPB2 or CERCAM had poorer OS comparing with low expressed groups (logrank = 0.0073, 0.0053, 0.0036 and 0.0023, respectively). Moreover, we also built a prognostic model using these five genes. The risk score of each sample was computed based on expression value of the 5 genes using cox proportional hazard model. High risk and low risk groups were divided by the best cutoff point of risk score (Supplementary Figure 2A, upper graph: the distribution of risk scores; lower graph: cutoff point selection based on log rank statistics). Survival analysis results showed that colorectal cancer patients with high risk score had poorer OS comparing with low risk groups (Supplementary Figure 2B, logrank p = 0.0016).

**Figure 3:**
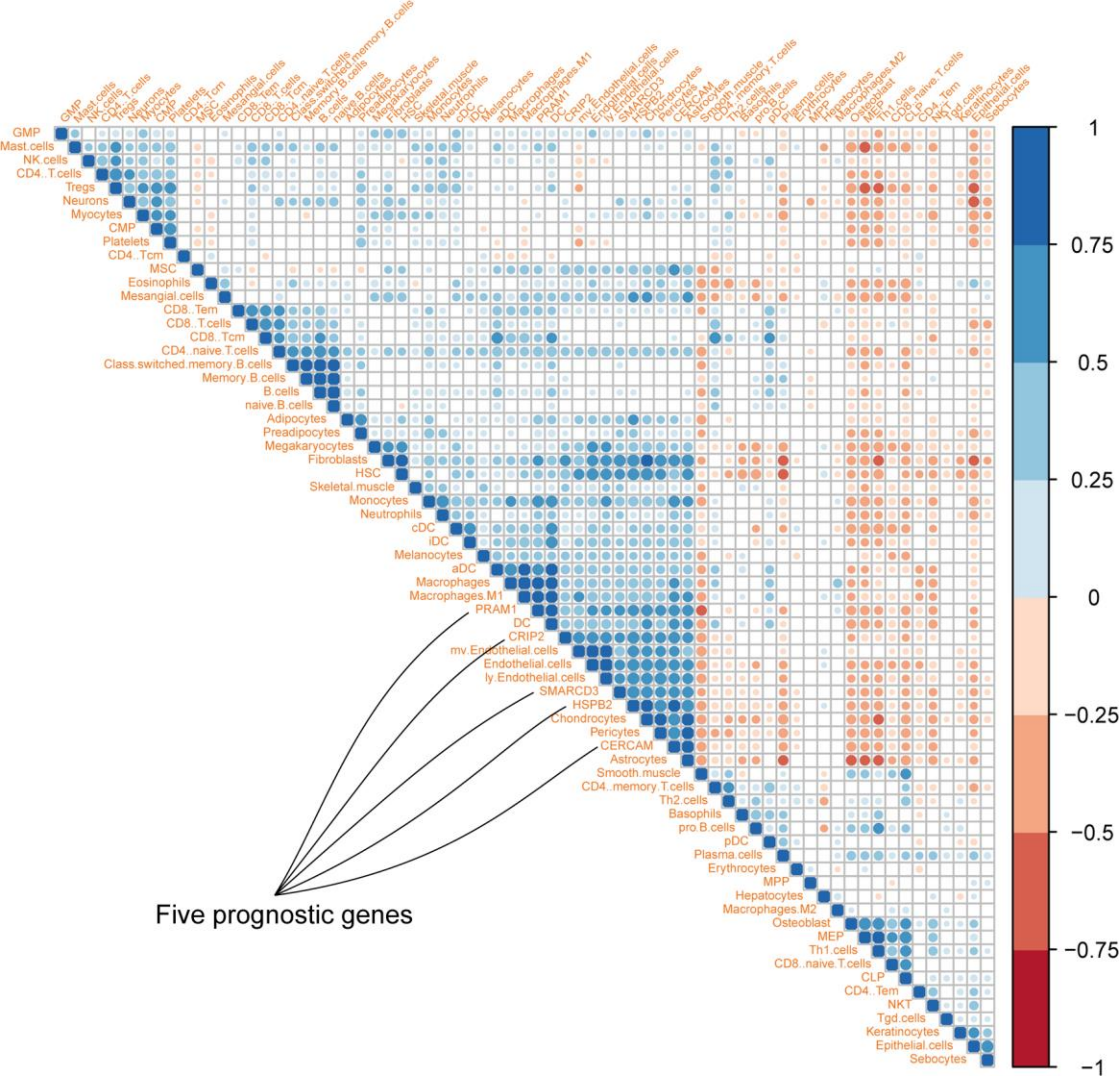
**Correlation map of five genes with different cell types in colon cancer microenvironment (Different colors represent spearman r values).**

To explore the association among these fives genes and different cell types in the tumor microenvironment, cellular heterogeneity analyses of tumor microenvironment were performed using xCell using ssGSEA method. The correlation map of the expression value of five genes and enrichment score of different cell components in TME were shown in Figure 3. As we can see, CRIP2 and PRAM1 are correlated with macrophages while SMARCD3, HSPB2 and CERCAM are associated with fibroblasts (blue represents positive correlation while red represents negative correlation, correlations with p value < 0.05 were presented in the map).

**Figure 4:**
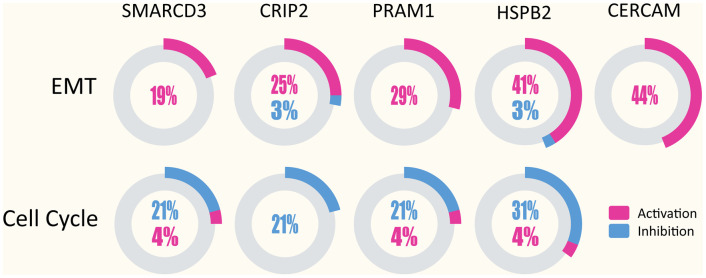
**SMARCD3, CRIP2, PRAM1, HSPB2 and CERCAM could activate EMT in multiple cancer types (upper panel), while SMARCD3, CRIP2, PRAM1 and HSPB2 are associated with cell cycle inhibition (lower panel).**

**Figure 5:**
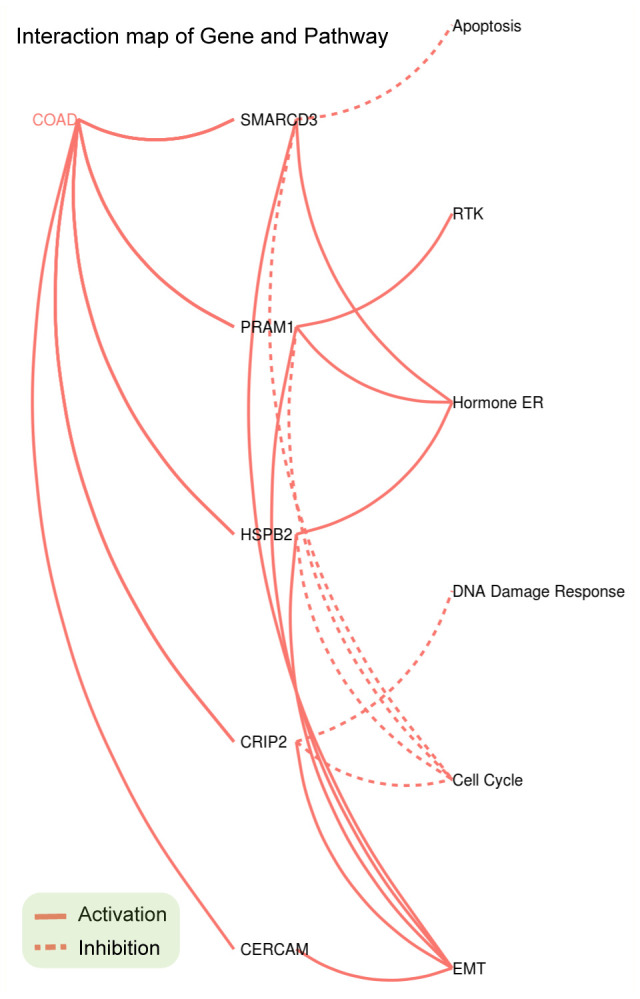
**Gene-Pathway interaction network of SMARCD3, CRIP2, PRAM1, HSPB2 and CERCAM in colorectal cancer.**

Pathway enrichment analyses of the five genes were performed using GSCAlite. As mentioned in the methods section, pathway activity scores (PAS) of 10 cancer related pathways (such as EMT, apoptosis and cell cycle etc.) in 32 cancer types were computed based on RPPA protein data from TCGA. PAS (gene X^high^) > PAS (gene X^low^) indicates gene X has an activation effect, otherwise an inhibition effect. Analyses results indicated that SMARCD3, CRIP2, PRAM1, HSPB2 and CERCAM were associated with epithelial-mesenchymal transition (EMT) pathway activation (upper panel of Figure 4) and cell cycle inhibition (lower panel of Figure 4). Percentage represents ratios of activation or inhibition related cancer types versus 32 cancer types. For instance, SMARCD3 has an activation effect in 6 over 32 cancer types (approximately 19%). Figure 5 shows the gene-pathway interaction map of these five genes in colon cancer. As we can see, SMARCD3 is associated with cell cycle inhibition and EMT activation, which are in accordance with Figure 4. Moreover, PPI network of these five genes was presented in Supplementary Figure 3.

### SMARCD3 expression and function analyses

**Figure 6:**
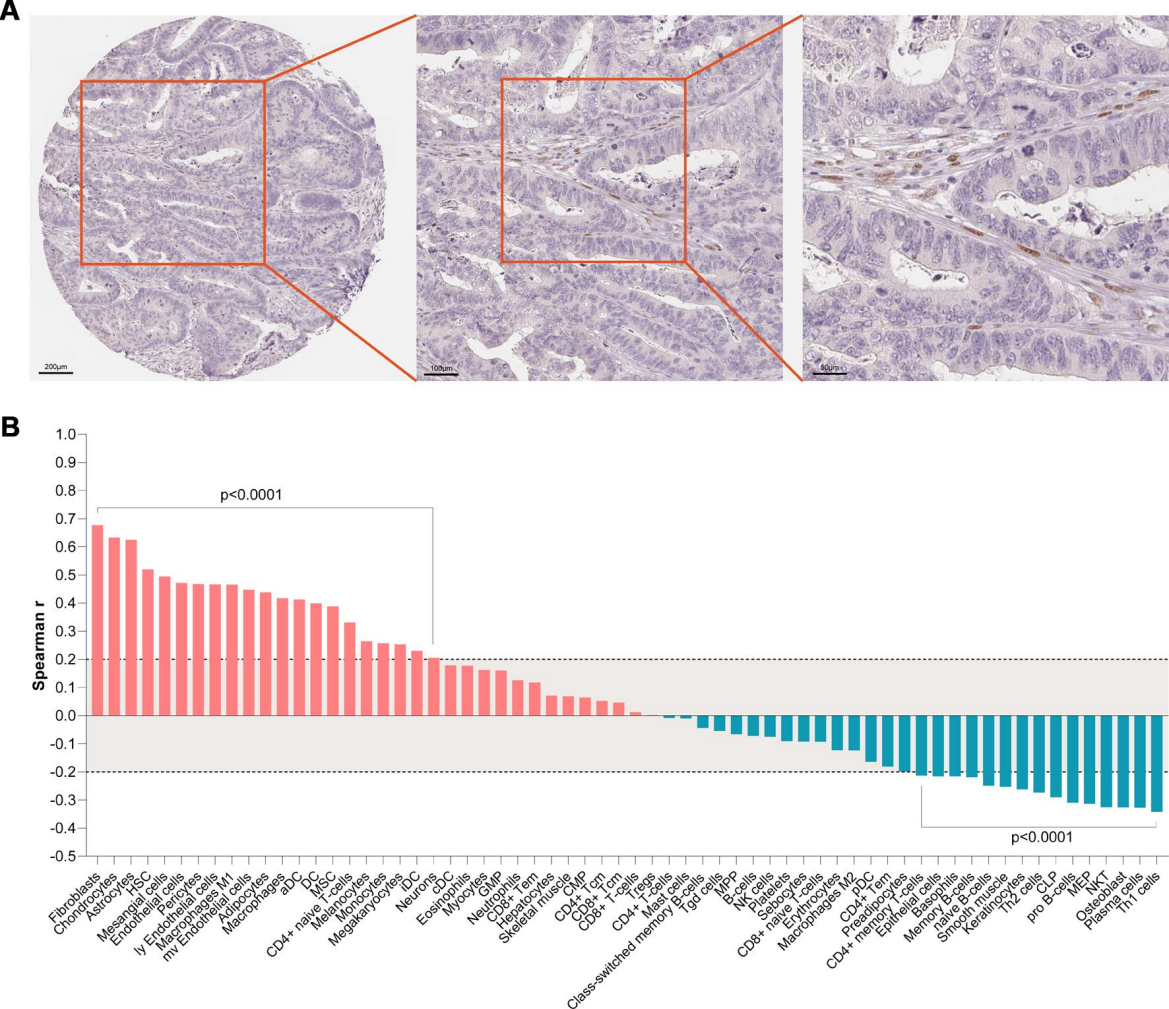
(**A**) IHC staining indicated that SMARCD3 was mainly expressed colon cancer associated fibroblasts. (**B**) SMARCD3 expression was most associated with fibroblasts by ssGSEA analysis.

Based on expression value and literature reports, SMARCD3 were selected for further analysis. Gene expression data analyses based on colon cancer data from TCGA indicated that SMARCD3 is under expressed in cancer tissues comparing with normal control (Supplementary Figure 4A). SMARCD3 expression is higher in stage 3 in comparison with stage 1 (Supplementary Figure 4B). There is no statistical difference of SMARCD3 expression among different gender, body weight, sample type, age groups and TP53 mutation status (Supplementary Figure 4C–4G). Its expression in N2 (positive lymph node between 4 and 9) is higher than in N0 (data not shown). Interestingly, SMARCD3 expression in primary colon tumor is higher than in polyps. Its expression in both polyps and primary tumor is significantly lower than in normal control, which may due to different methylation levels at its promoter region (Supplementary Figure 5). Correlation analyses using SMARCD3 expression data and clinical features of colon cancer patients from TCGA indicated that SMARCD3 expression is associated with lymphatic invasion, OS and copy number etc. (Supplementary Figure 6). The prognostic value of SMARCD3 was further validated using a larger set of TCGA colorectal cancer RNAseq data. As was shown in Supplementary Figure 7, SMARCD3 expression was negatively correlated OS (logrank p = 0.0005, Hazard ratio = 1.867, N = 597), which was consistent with Figure 2B. The prognostic power of SMARCD3 is inferior to the five gene prognostic model presented in Supplementary Figure 2B. IHC data from the protein atlas showed that SMARCD3 were mainly expressed in fibroblasts (Figure 6A). Correlation analysis indicated that SMARCD3 expression was most correlated with fibroblasts (Figure 6B), which was in accordance with Figure 6A.

**Figure 7:**
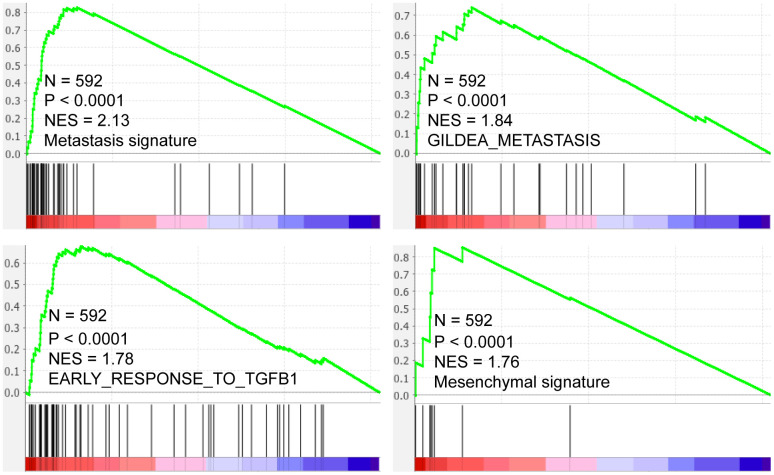
**SMARCD3 expression is positively correlated with metastasis, TGF-β pathway activation and Mesenchymal signatures.**

**Figure 8:**
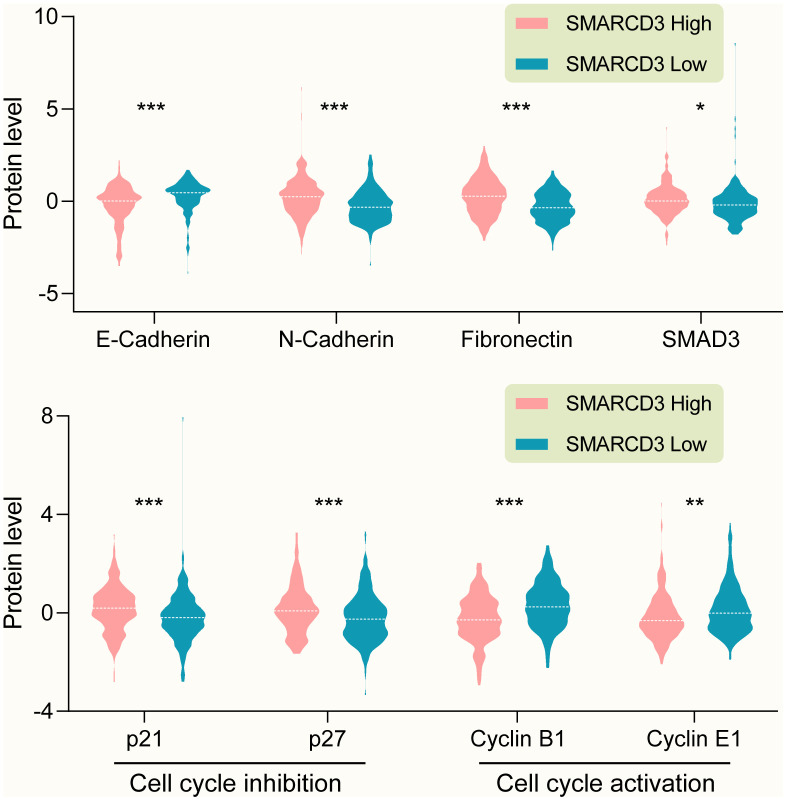
**(Upper graph) SMARCD3 expression is negatively correlated with E-Cadherin, while positively correlated with N-Cadherin, Fibronectin and SMAD3. (Lower graph) SMARCD3 expression is positively correlated with cell cycle inhibitor p21 and p27, while negatively correlated with cell cycle activator Cyclin B1 and Cyclin E1.**

GSEA results showed that SMARCD3 expression was associated with cancer metastasis, TGF-β pathway activation and epithelial-mesenchymal transition (EMT) (Figure 7, p < 0.0001, TCGA colorectal cancer RNAseq data, N = 592). Protein level analyses indicated that SMARCD3 expression was negatively correlated with E-Cadherin while positively correlated with N-Cadherin, Fibronectin and SMAD3 (Figure 8, upper graph), which further proved its association with EMT. We also showed that SMARCD3 expression was positively correlated with cell cycle inhibition markers such as p21 an p27, while negatively correlated with cell cycle activation markers such as Cyclin B1 and Cyclin E1 (Figure 8, lower graph). PPI network analysis demonstrated that SMARCD3 could physically interacted with MAPK14 (p38α), MYOD1 and SMAD4 etc. (Supplementary Figure 8), which indicated mechanistic insights underlying SMARCD3 related colorectal cancer metastasis.

### Potential molecular mechanisms underlying SMARCD3 associated cancer metastasis

As we mentioned in the above section, SMARCD3 was associated with cancer metastasis and EMT related gene signatures. Further PPI analysis indicated that SMARCD3 might promote colorectal cancer metastasis through MAPK14, MYOD1 or SMAD4 related pathways. Previously, it was reported that SMARCD3 could stimulate EMT of breast cancer cells through upregulating WNT5A expression [11]. While Wnt5a and Wnt11 could regulate EMT by inducing p38 (Mapk14) phosphorylation in mouse early development [12]. Hence, we can speculate that SMARCD3 could potentially promote EMT through WNT5A-MAPK14 pathway. Moreover, it was reported that MAPK14 could induce SMARCD3 phosphorylation and promote the incorporation of MYOD1-SMARCD3 into a Brg1-based SWI/SNF complex. This complex could activate the transcription activity of MYOD1 [13] and led to upregulation of EMT related genes such as Vimentin and SNAIL [14]. These reports indicated that MAPK14 could regulate EMT by phosphorylating SMARCD3. So, we can conclude that there is a positive feedback loop among SMARCD3, WNT5A and MAPK14. Moreover, it was reported that TGF-β could also promote EMT through MAPK14 phosphorylation [15], which indicated its involvement in the process of SMARCD3 promoted EMT.

**Figure 9:**
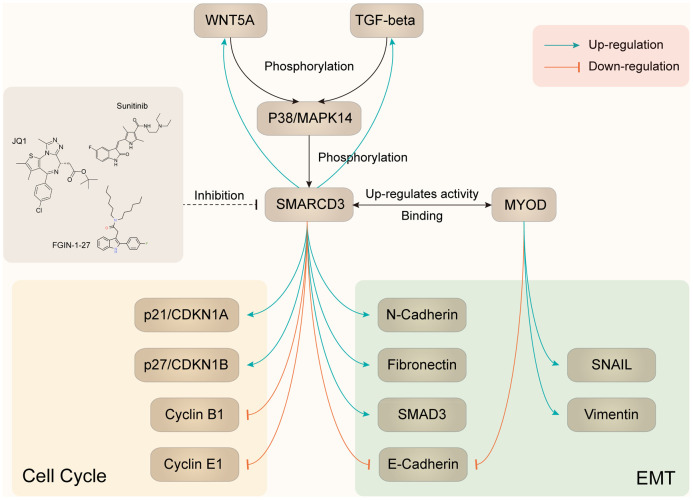
**Summary of SMARCD3 interaction network.**

Using TCGA colorectal cancer RNAseq and protein expression data, we demonstrated that WNT5A (Supplementary Figure 9A) and TGFB1 (Supplementary Figure 9C) were positively correlated with SMARCD3; WNT5A (Supplementary Figure 9B) and TGFB1 (Supplementary Figure 9D) were overexpressed in SMARCD3 high group. We also showed that SMARCD3 was associated with MAPK14 phosphorylation level (Supplementary Figure 10). The above results were consistent with the above literature reports and our speculations. Based on the above results, we could summarize two potential positive feedback loops: SMARCD3-WNT5A-MAPK14-SMARCD3 and SMARCD3-TGF-β-MAPK14-SMARCD3 (Figure 9). Besides, data mining using multiple gene-drug datasets such as CTDbase and GSCAlite were performed in this study. We proposed several drugs that could target SMARCD3, which were presented in Figure 9.

## DISCUSSION

In this study, we found 372 genes that overexpressed in TME based on immune score and stromal score using TCGA COAD data from two platforms (Agilent and RNAseq). GO and KEGG pathway enrichment analyses showed that these 372 genes were enriched in immune response, cytokine production and toll-like receptor signaling pathway etc.. SMARCD3, CRIP2, PRAM1, HSPB2 and CERCAM were selected for further analyses due to their most significant prognostic powers. Cellular heterogeneity analysis indicated that PRAM1 was associated with macrophages while SMARCD3, CRIP2, HSPB2 and CERCAM were correlated with fibroblasts. Pathway analyses showed that the five genes were involved in EMT activation and cell cycle inhibition. Since EMT was an important factor for CAFs activation, the above results implicated a potential role of these genes in inducing CAFs activation.

CAFs plays critical roles in tumor proliferation, invasion, angiogenesis and regulation of tumor immune microenvironment. For instance, CAFs could release exosomes, VEGF (vascular endothelial growth factor), HGF (hepatocyte growth factor) and GAS6 (growth arrest-specific gene 6) to promote cancer proliferation and invasion, and affect the function of epithelial cell and macrophages [16]; regulate cancer metastasis, therapeutic responses and T cell function through matrix remodeling including matrix production, proteolysis and matrix crosslinking [17, 18]; promote cancer cell growth through metabolic effects such as lactate shuttling, amino acid depletion and alanine aspartate shuttling [19, 20]; and regulate cancer immune microenvironment through TGF-β, IL-6, CXCL12 (CXC- chemokine ligand 12) and CCL2 (CC-chemokine ligand 2) [21–23].

SMARCD3 (SWI/SNF Related, Matrix Associated, Actin Dependent Regulator of Chromatin, Subfamily D, Member 3) encoded protein belongs to SWI/SNF family which display helicase and ATPase activities and could regulate gene transcription by altering the chromatin. It was reported that knock down SMARCD3 expression could induce mesenchymal-epithelial transition (EMT) of breast cancer cells [24]. Here, we showed that SMARCD3 was mainly expressed in fibroblasts and was associated with EMT and tumor metastasis. Its expression was positively correlated with mesenchymal biomarkers such as N-Cadherin and Fibronectin while negatively correlated with epithelial biomarkers like E-Cadherin. Literature mining indicated that SMARCD3 could upregulate WNT5A and TGF-β expression, which could induce MAPK14 phosphorylation. Then the phosphorylated MAPK14 could further induce SMARCD3 phosphorylation and promote the incorporation of MYOD1-SMARCD3 into a Brg1-based SWI/SNF complex and finally led to EMT. Using colorectal cancer data, we showed that SMARCD3 expression was positively correlated with WNT5A, TGF-β and p-MAPK14, which were consistent with previous reports.

Based on the above findings, we speculated that SMARCD3-WNT5A/TGF-β-MAPK14-SMARCD3 positive feedback loop might be activated in fibroblasts and play critical roles in promoting CAFs activation and cancer metastasis (as detailed in Figure 9).

In summary, we reported 372 colorectal cancer TME related genes, five of them that have the most prognostic powers were enriched in EMT and cell cycle pathways. For the first time, we demonstrated that SMARCD3 was a novel prognostic marker that mainly expressed in CAFs and might promote CAFs activation and colorectal cancer metastasis through SMARCD3-WNT5A/TGF-β-MAPK14-SMARCD3 positive feedback loop. Hence, screening for drugs or chemicals targeting SMARCD3 may exert important clinical impact on colorectal cancer management.

## MATERIALS AND METHODS

### Ethics statement

All the data used in this study were downloaded from publicly available sources. The Research Ethics Committee of Zhejiang Provincial people’s Hospital waived the requirement for ethical approval.

### Data source

Agilent microarray and RNAseq expression data were downloaded from The Cancer Genome Atlas (TCGA: http://cancergenome.nih.gov/). Expression profiles of colon, polyp and primary colon cancer were obtained from Gene Expression Omnibus (GEO, accession no.GSE41258) [25, 26]. IHC staining results of SMARCD3 (https://images.proteinatlas.org/63955/147563_A_2_8.jpg), Protein expression and phosphorylation data were obtained from Protein atlas [27] (https://www.proteinatlas.org) and TCGA. Chemical-gene interaction and protein-protein interaction data was downloaded from The Comparative Toxicogenomics Database (CTD base) [28] and GSCAlite [29].

### Bioinformatics and statistical analyses

The immune score and stromal score of each colon cancer samples were computed based on ESTIMATE algorithm using RNAseq data from TCGA [30]. Heat map and clustering analyses were performed using MeV software (http://mev.tm4.org). Gene Set Enrichment analysis (GSEA) was performed to show the functional enrichment of SMARCD3 in breast cancer using GSEA v4.0.3 (https://www.gsea-msigdb.org/gsea/downloads.jsp). Protein-protein interaction network was visualized through GeneMANIA plugin [31] in the Cytoscape environment [32]. Venn diagram was drawn using an online tool (http://bioinformatics.psb.ugent.be/webtools/Venn/). GO and KEGG pathway enrichment analyses and visualization were performed using NetworkAnalyst [33]. Survival analysis module of GEPIA2 web tool and Graphpad Prism 8 (2365 Northside Dr., Suite 560, San Diego, CA 92108, USA) was used for Kaplan-Meier analyses [34].

Gene-pathway interaction network analysis was performed using GSCAlite [29]. Briefly, pathway activity groups (activation and inhibition) is defined by pathway scores computed based on RPPA protein data from TCGA, 10 pathways and 32 cancer types are included. Gene expression positively correlated with pathway activity score are considered to have an activate effect to a pathway, otherwise have an inhibit effect to a pathway. Cellular heterogeneity analyses of tumor microenvironment were performed using xCell using ssGSEA method [35]. Correlation map was drew using corrPlot package [36] in R 3.6.3 (R Foundation for Statistical Computing [http://www.r-project.org/]). Expression of SMARCD3 in different clinical groups and its correlation with methylation were plotted using UALCAN based on data from TCGA [37]. The heat map of SMARCD3 expression and clinical features such as tumor stage, lymphatic invasion and overall survival was plotted using MEXPRESS online tool [38].

Risk score of each sample was computed based on expression value of the 5 genes using cox proportional hazard model. The best cutoff value of 5 gene risk score was computed using survminer package (https://rpkgs.datanovia.com/survminer/index.html) and Kaplan-Meier analyses were performed through survival package [39] in R. All other statistical analyses were perform using R or GraphPad Prism 8. Standard statistical tests including paired t-test, fisher exact test and independent samples t-test were employed in the data analyses. Adjust P value was corrected for multiple comparisons using the Benjamini and Hochberg's false discovery rate [40]. Significance was defined as a P value < 0.05.

### Availability of data

Data sharing is not applicable to this article as no new data were created or analyzed in this study.

## Supplementary Material

Supplementary Table 2

Supplementary Table 1

Supplementary Table 6

Supplementary Tables 7 and 8

Supplementary Tables 3, 4 and 5

Supplementary Figures
