# Supplementary material for: SMARCD3 is a potential prognostic marker and therapeutic target in CAFs
Source: Aging (Albany NY). 2020 Oct 28;12(20):20835–61. doi: 10.18632/aging.104102 (PMC7655158; doi:10.18632/aging.104102)
Supplement: Supplementary Tables 7 and 8 [file aging-12-104102-s004..pdf]

SUPPLEMENTARY TABLES

Supplementary Table 7. Pearson correlation of SMARCD3 expression with different cell types in TME.

Cell type	Spearman r	95% confidence interval	P (two-tailed)	P value summary	Exact or approximate P value?	Significant? (alpha = 0.05)
Fibroblasts	0.6773	0.6158 to 0.7306	<0.0001	****	Approximate	Yes
Chondrocytes	0.6335	0.5658 to 0.6927	<0.0001	****	Approximate	Yes
Astrocytes	0.6248	0.5559 to 0.6851	<0.0001	****	Approximate	Yes
HSC	0.52	0.4385 to 0.5930	<0.0001	****	Approximate	Yes
Mesangial cells	0.4945	0.4104 to 0.5702	<0.0001	****	Approximate	Yes
Endothelial cells	0.472	0.3857 to 0.5501	<0.0001	****	Approximate	Yes
Pericytes	0.4678	0.3811 to 0.5463	<0.0001	****	Approximate	Yes
ly Endothelial cells	0.4661	0.3793 to 0.5448	<0.0001	****	Approximate	Yes
Macrophages M1	0.4653	0.3783 to 0.5441	<0.0001	****	Approximate	Yes
mv Endothelial cells	0.4469	0.3583 to 0.5275	<0.0001	****	Approximate	Yes
Adipocytes	0.4385	0.3492 to 0.5200	<0.0001	****	Approximate	Yes
Macrophages	0.4171	0.3259 to 0.5006	<0.0001	****	Approximate	Yes
aDC	0.4127	0.3211 to 0.4966	<0.0001	****	Approximate	Yes
DC	0.3992	0.3066 to 0.4843	<0.0001	****	Approximate	Yes
MSC	0.3889	0.2956 to 0.4750	<0.0001	****	Approximate	Yes
CD4+ naive T-cells	0.331	0.2336 to 0.4218	<0.0001	****	Approximate	Yes
Melanocytes	0.2645	0.1635 to 0.3600	<0.0001	****	Approximate	Yes
Monocytes	0.2571	0.1558 to 0.3531	<0.0001	****	Approximate	Yes
Megakaryocytes	0.2538	0.1524 to 0.3499	<0.0001	****	Approximate	Yes
iDC	0.23	0.1276 to 0.3276	<0.0001	****	Approximate	Yes
Neurons	0.2055	0.1022 to 0.3044	<0.0001	****	Approximate	Yes
cDC	0.1788	0.07472 to 0.2791	0.0006	***	Approximate	Yes
Eosinophils	0.1776	0.07346 to 0.2779	0.0006	***	Approximate	Yes
Myocytes	0.162	0.05742 to 0.2630	0.0019	**	Approximate	Yes
GMP	0.1603	0.05569 to 0.2614	0.0021	**	Approximate	Yes
Neutrophils	0.1258	0.02056 to 0.2283	0.016	*	Approximate	Yes
CD8+ Tem	0.1173	0.01194 to 0.2201	0.0248	*	Approximate	Yes
Hepatocytes	0.07119	-0.03459 to 0.1754	0.1741	ns	Approximate	No
Skeletal muscle	0.06895	-0.03684 to 0.1732	0.1881	ns	Approximate	No
CMP	0.06416	-0.04164 to 0.1685	0.2208	ns	Approximate	No
CD4+ Tcm	0.052	-0.05381 to 0.1567	0.3212	ns	Approximate	No
CD8+ Tcm	0.04653	-0.05928 to 0.1513	0.3748	ns	Approximate	No
CD8+ T-cells	0.0126	-0.09304 to 0.1180	0.8102	ns	Approximate	No
Tregs	0.00172	-0.1038 to 0.1072	0.9738	ns	Approximate	No
CD4+ T-cells	-0.008136	-0.1136 to 0.09747	0.8767	ns	Approximate	No
Mast cells	-0.01023	-0.1156 to 0.09539	0.8454	ns	Approximate	No
Class-switched memory B-cells	-0.04365	-0.1485 to 0.06215	0.405	ns	Approximate	No
Tgd cells	-0.05474	-0.1593 to 0.05107	0.2963	ns	Approximate	No
MPP	-0.06603	-0.1704 to 0.03976	0.2075	ns	Approximate	No
B-cells	-0.0714	-0.1756 to 0.03438	0.1729	ns	Approximate	No
NK cells	-0.07455	-0.1787 to 0.03121	0.1546	ns	Approximate	No

Platelets	-0.09096	-0.1946 to 0.01469	0.0822	ns	Approximate	No
Sebocytes	-0.09207	-0.1957 to 0.01358	0.0786	ns	Approximate	No
CD8+ naive T-cells	-0.09239	-0.1960 to 0.01326	0.0775	ns	Approximate	No
Erythrocytes	-0.123	-0.2256 to -0.01773	0.0185	*	Approximate	Yes
Macrophages M2	-0.1236	-0.2262 to -0.01834	0.018	*	Approximate	Yes
pDC	-0.1642	-0.2652 to -0.05975	0.0016	**	Approximate	Yes
CD4+ Tem	-0.1811	-0.2812 to -0.07703	0.0005	***	Approximate	Yes
Preadipocytes	-0.1983	-0.2976 to -0.09473	0.0001	***	Approximate	Yes
CD4+ memory T-cells	-0.2133	-0.3118 to -0.1103	<0.0001	****	Approximate	Yes
Epithelial cells	-0.2158	-0.3142 to -0.1129	<0.0001	****	Approximate	Yes
Basophils	-0.2161	-0.3145 to -0.1132	<0.0001	****	Approximate	Yes
Memory B-cells	-0.2192	-0.3173 to -0.1163	<0.0001	****	Approximate	Yes
naive B-cells	-0.2487	-0.3452 to -0.1471	<0.0001	****	Approximate	Yes
Smooth muscle	-0.253	-0.3492 to -0.1515	<0.0001	****	Approximate	Yes
Keratinocytes	-0.2623	-0.3579 to -0.1612	<0.0001	****	Approximate	Yes
Th2 cells	-0.2733	-0.3682 to -0.1727	<0.0001	****	Approximate	Yes
CLP	-0.2904	-0.3842 to -0.1907	<0.0001	****	Approximate	Yes
pro B-cells	-0.3101	-0.4025 to -0.2115	<0.0001	****	Approximate	Yes
MEP	-0.3131	-0.4052 to -0.2146	<0.0001	****	Approximate	Yes
NKT	-0.3256	-0.4168 to -0.2279	<0.0001	****	Approximate	Yes
Osteoblast	-0.3262	-0.4174 to -0.2286	<0.0001	****	Approximate	Yes
Plasma cells	-0.3272	-0.4183 to -0.2296	<0.0001	****	Approximate	Yes
Th1 cells	-0.3425	-0.4324 to -0.2459	<0.0001	****	Approximate	Yes

Supplementary Table 8. KEGG pathway analysis of SMARCD3 Protein-Protein network.

Pathway	Total	Expected	Hits	P.Value	FDR
Transcriptional misregulation in cancer	186	0.769	8	6.16E-07	0.000196
Th17 cell differentiation	107	0.443	6	4.11E-06	0.000654
Wnt signaling pathway	158	0.653	5	0.00043	0.0415
Th1 and Th2 cell differentiation	92	0.381	4	0.000522	0.0415
Endocrine resistance	98	0.405	4	0.000663	0.0422
Pathways in cancer	530	2.19	8	0.00111	0.0567
Thyroid hormone signaling pathway	116	0.48	4	0.00125	0.0567
Osteoclast differentiation	128	0.529	4	0.0018	0.0662
HTLV-I infection	219	0.906	5	0.00187	0.0662
Non-alcoholic fatty liver disease (NAFLD)	149	0.616	4	0.00312	0.0993
AGE-RAGE signaling pathway in diabetic complications	100	0.414	3	0.00792	0.229
Epstein-Barr virus infection	201	0.831	4	0.00899	0.238
Thyroid cancer	37	0.153	2	0.0101	0.247
Notch signaling pathway	48	0.199	2	0.0166	0.377
Estrogen signaling pathway	138	0.571	3	0.0189	0.382
Signaling pathways regulating pluripotency of stem cells	139	0.575	3	0.0192	0.382
Breast cancer	147	0.608	3	0.0223	0.417
Hepatitis B	163	0.674	3	0.0291	0.455
Inflammatory bowel disease (IBD)	65	0.269	2	0.0293	0.455
Non-small cell lung cancer	66	0.273	2	0.0302	0.455
Epithelial cell signaling in Helicobacter pylori infection	68	0.281	2	0.0319	0.455
MAPK signaling pathway	295	1.22	4	0.0321	0.455
Prolactin signaling pathway	70	0.29	2	0.0336	0.455
Bile secretion	72	0.298	2	0.0354	0.455
PPAR signaling pathway	74	0.306	2	0.0372	0.455
Leishmaniasis	74	0.306	2	0.0372	0.455
Pertussis	76	0.314	2	0.0391	0.46
Salmonella infection	86	0.356	2	0.0488	0.525
Colorectal cancer	86	0.356	2	0.0488	0.525
