# Supplementary material for: SMARCD3 is a potential prognostic marker and therapeutic target in CAFs
Source: Aging (Albany NY). 2020 Oct 28;12(20):20835–61. doi: 10.18632/aging.104102 (PMC7655158; doi:10.18632/aging.104102)
Supplement: Supplementary Tables 3, 4 and 5 [file aging-12-104102-s005..pdf]

SUPPLEMENTARY TABLES

Supplementary Table 3. GO analysis of 372 genes (Cellular Component).

Pathway	Total	Expected	Hits	P.Value	FDR
Integral to plasma membrane	1270	26.3	79	2.82E-19	3.32E-17
External side of plasma membrane	204	4.22	32	2.95E-19	3.32E-17
Intrinsic to plasma membrane	1320	27.4	80	8.66E-19	6.49E-17
Plasma membrane part	2320	47.9	109	1.30E-17	7.30E-16
Cell surface	518	10.7	45	2.80E-16	1.26E-14
Plasma membrane	5500	114	185	1.28E-15	4.78E-14
Extracellular space	901	18.6	57	3.16E-14	1.02E-12
Extracellular region part	1320	27.3	71	5.00E-14	1.40E-12
Intrinsic to membrane	5760	119	182	2.11E-12	5.26E-11
Integral to membrane	5590	116	178	2.34E-12	5.26E-11
Lytic vacuole	401	8.3	29	5.34E-09	1.00E-07
Lysosome	401	8.3	29	5.34E-09	1.00E-07
Extracellular matrix	570	11.8	32	3.27E-07	5.53E-06
Vacuole	486	10.1	29	3.44E-07	5.53E-06
Extracellular region	2860	59.1	96	4.05E-07	6.08E-06
Membrane part	7520	156	201	5.16E-07	7.25E-06
Proteinaceous extracellular matrix	398	8.24	23	9.83E-06	0.00013
Collagen	93	1.92	10	2.23E-05	0.000279
Receptor complex	189	3.91	14	4.04E-05	0.000479
Vacuolar part	279	5.77	17	7.50E-05	0.000844
Extracellular matrix part	204	4.22	12	0.00115	0.012
Immunological synapse	23	0.476	4	0.00117	0.012
Lysosomal membrane	153	3.17	10	0.00133	0.013
Membrane raft	189	3.91	11	0.00198	0.0173
Ruffle	135	2.79	9	0.002	0.0173
Extrinsic to membrane	135	2.79	9	0.002	0.0173
Extrinsic to plasma membrane	87	1.8	7	0.00217	0.0181
Anchored to membrane	147	3.04	9	0.00355	0.0285
Actin filament	51	1.06	5	0.00397	0.0308
Integrin complex	32	0.662	4	0.0041	0.0308
Anchored to plasma membrane	33	0.683	4	0.00459	0.0333
Endocytic vesicle	187	3.87	10	0.00567	0.0398
Vacuolar membrane	206	4.26	10	0.0109	0.074
Vesicle	1210	25	34	0.0422	0.279
Membrane_bounded vesicle	1100	22.7	31	0.0493	0.317

Supplementary Table 4. GO analysis of 372 genes (Molecular Function).

Pathway	Total	Expected	Hits	P.Value	FDR
Receptor activity	1920	38.1	79	8.32E-11	3.23E-08
Carbohydrate binding	227	4.5	21	4.83E-09	6.25E-07
Carbohydrate binding	227	4.5	21	4.83E-09	6.25E-07
Cytokine activity	220	4.36	18	4.00E-07	3.88E-05
Glycosaminoglycan binding	178	3.53	15	2.61E-06	0.000203
Pattern recognition receptor activity	17	0.337	5	1.50E-05	0.000972
Cytokine receptor binding	266	5.27	17	2.32E-05	0.00124
Transmembrane signaling receptor activity	1510	30	53	2.56E-05	0.00124
Cytokine receptor activity	106	2.1	10	4.76E-05	0.00185
Cytokine receptor activity	106	2.1	10	4.76E-05	0.00185
Chemokine activity	50	0.991	7	5.38E-05	0.0019
Chemokine receptor binding	75	1.49	8	0.000115	0.00373
Cytokine binding	66	1.31	7	0.00032	0.00887
Cytokine binding	66	1.31	7	0.00032	0.00887
Protein complex binding	339	6.72	17	0.000441	0.0114
Receptor binding	1590	31.6	49	0.00118	0.0286
Lipoprotein particle binding	26	0.515	4	0.0016	0.0365
Antigen binding	66	1.31	6	0.00193	0.0412
Lipid binding	788	15.6	28	0.00202	0.0412
Collagen binding	47	0.931	5	0.00229	0.0445
Enzyme activator activity	436	8.64	18	0.00275	0.0508
Low_density lipoprotein particle binding	15	0.297	3	0.00294	0.0518
Phospholipid binding	514	10.2	19	0.00694	0.117
Metalloexopeptidase activity	42	0.832	4	0.00936	0.151
Peptide receptor activity	150	2.97	8	0.0101	0.157
Non_membrane spanning protein tyrosine kinase activity	46	0.912	4	0.0128	0.192
Identical protein binding	910	18	28	0.0138	0.198
Heparin binding	130	2.58	7	0.0149	0.207
GTP binding	371	7.35	14	0.0163	0.214
Antioxidant activity	75	1.49	5	0.0165	0.214
G_protein coupled receptor binding	232	4.6	10	0.0175	0.219
Protein homodimerization activity	573	11.4	19	0.02	0.243
Extracellular matrix structural constituent	80	1.59	5	0.0213	0.25
Kinase regulator activity	144	2.85	7	0.0247	0.282
Guanyl nucleotide binding	402	7.97	14	0.0298	0.33
Growth factor binding	125	2.48	6	0.0382	0.407
Protein dimerization activity	996	19.7	28	0.0388	0.407
Phosphatidylinositol binding	160	3.17	7	0.0403	0.411
Rho GTPase activator activity	39	0.773	3	0.0417	0.415
Phospholipase C activity	41	0.813	3	0.0473	0.458

Supplementary Table 5. KEGG pathway analysis of 372 genes.

Pathway	Total	Expected	Hits	P.Value	FDR
Osteoclast differentiation	128	3.23	31	1.52E-22	4.85E-20
Staphylococcus aureus infection	68	1.71	18	3.24E-14	5.16E-12
Tuberculosis	179	4.51	22	5.63E-10	5.97E-08
Leishmaniasis	74	1.87	14	3.17E-09	2.52E-07
Toll-like receptor signaling pathway	104	2.62	16	5.43E-09	3.32E-07
Rheumatoid arthritis	91	2.29	15	6.27E-09	3.32E-07
Hematopoietic cell lineage	97	2.44	15	1.55E-08	7.03E-07
Cytokine-cytokine receptor interaction	294	7.41	25	7.67E-08	3.05E-06
Chemokine signaling pathway	190	4.79	19	2.61E-07	9.21E-06
Pertussis	76	1.92	12	3.54E-07	1.13E-05
Chagas disease (American trypanosomiasis)	103	2.6	13	1.63E-06	4.70E-05
Malaria	49	1.23	9	2.93E-06	7.77E-05
Complement and coagulation cascades	79	1.99	11	3.98E-06	9.73E-05
Phagosome	152	3.83	15	5.92E-06	0.000135
Leukocyte transendothelial migration	112	2.82	12	2.28E-05	0.000483
Lysosome	123	3.1	12	5.82E-05	0.00116
Cell adhesion molecules (CAMs)	146	3.68	13	7.45E-05	0.00139
Intestinal immune network for IgA production	49	1.23	7	0.000203	0.00359
B cell receptor signaling pathway	71	1.79	8	0.000385	0.00645
Legionellosis	55	1.39	7	0.000422	0.00672
NOD-like receptor signaling pathway	178	4.49	13	0.000536	0.00811
NF-kappa B signaling pathway	100	2.52	9	0.000897	0.013
Influenza A	167	4.21	12	0.00101	0.014
Inflammatory bowel disease (IBD)	65	1.64	7	0.00117	0.0155
Natural killer cell mediated cytotoxicity	131	3.3	10	0.00168	0.0214
TNF signaling pathway	110	2.77	9	0.00176	0.0216
Systemic lupus erythematosus	133	3.35	10	0.00188	0.0222
Fc gamma R-mediated phagocytosis	91	2.29	8	0.002	0.0228
Toxoplasmosis	113	2.85	9	0.00213	0.0233
Measles	138	3.48	10	0.00248	0.0251
Kaposi's sarcoma-associated herpesvirus infection	186	4.69	12	0.00253	0.0251
Transcriptional misregulation in cancer	186	4.69	12	0.00253	0.0251
T cell receptor signaling pathway	101	2.55	8	0.00385	0.0371
Proteoglycans in cancer	201	5.07	12	0.00475	0.0445
Th17 cell differentiation	107	2.7	8	0.00547	0.0497
Acute myeloid leukemia	66	1.66	6	0.00615	0.0543
Th1 and Th2 cell differentiation	92	2.32	7	0.00835	0.0699
Fluid shear stress and atherosclerosis	139	3.5	9	0.00835	0.0699
Amoebiasis	96	2.42	7	0.0105	0.0852
Prion diseases	35	0.882	4	0.0111	0.0884
Antigen processing and presentation	77	1.94	6	0.0128	0.0978
AGE-RAGE signaling pathway in diabetic complications	100	2.52	7	0.0129	0.0978
Allograft rejection	38	0.958	4	0.0148	0.109
Graft-versus-host disease	41	1.03	4	0.0191	0.138

Jak-STAT signaling pathway	162	4.08	9	0.0211	0.149
Fc epsilon RI signaling pathway	68	1.71	5	0.028	0.194
IL-17 signaling pathway	93	2.34	6	0.0296	0.198
Sphingolipid signaling pathway	119	3	7	0.0304	0.198
Epstein-Barr virus infection	201	5.07	10	0.0305	0.198
RIG-I-like receptor signaling pathway	70	1.76	5	0.0312	0.199
PI3K-Akt signaling pathway	354	8.92	15	0.0335	0.209
Platelet activation	124	3.13	7	0.0369	0.225
Asthma	31	0.781	3	0.0422	0.253
Pathogenic Escherichia coli infection	55	1.39	4	0.0493	0.289
HTLV-I infection	219	5.52	10	0.0499	0.289
