# Supplementary material for: SMARCD3 is a potential prognostic marker and therapeutic target in CAFs
Source: Aging (Albany NY). 2020 Oct 28;12(20):20835–61. doi: 10.18632/aging.104102 (PMC7655158; doi:10.18632/aging.104102)
Supplement: Supplementary Figures [file aging-12-104102-s006..pdf]

SUPPLEMENTARY FIGURES

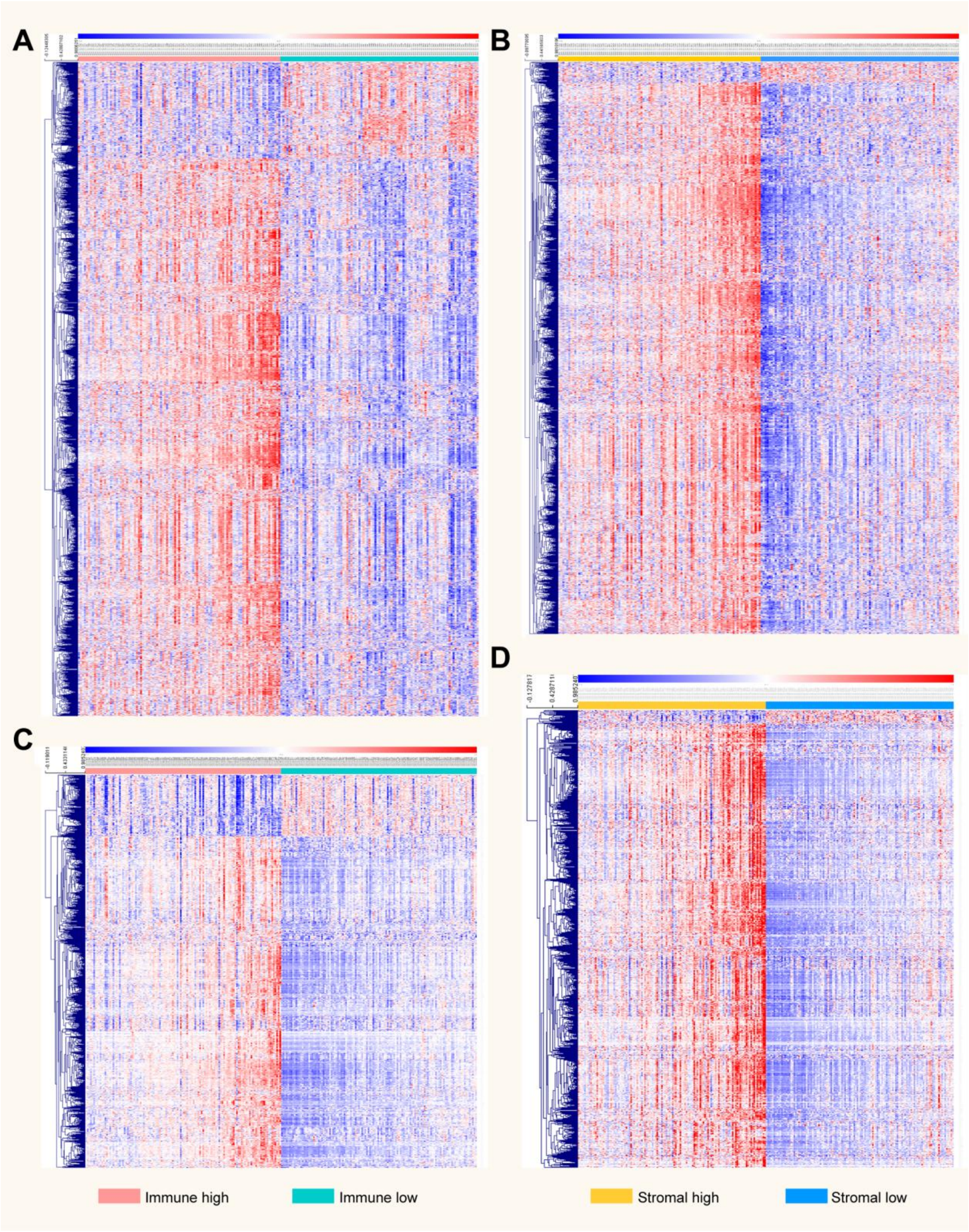

Supplementary Figure 1. Heat map of TME related genes. DEGs computed based on immune (**A**) or stromal score (**B**) using HNSC Agilent microarray data. DEGs computed based on immune (**C**) or stromal score (**D**) using HNSC RNAseq data.

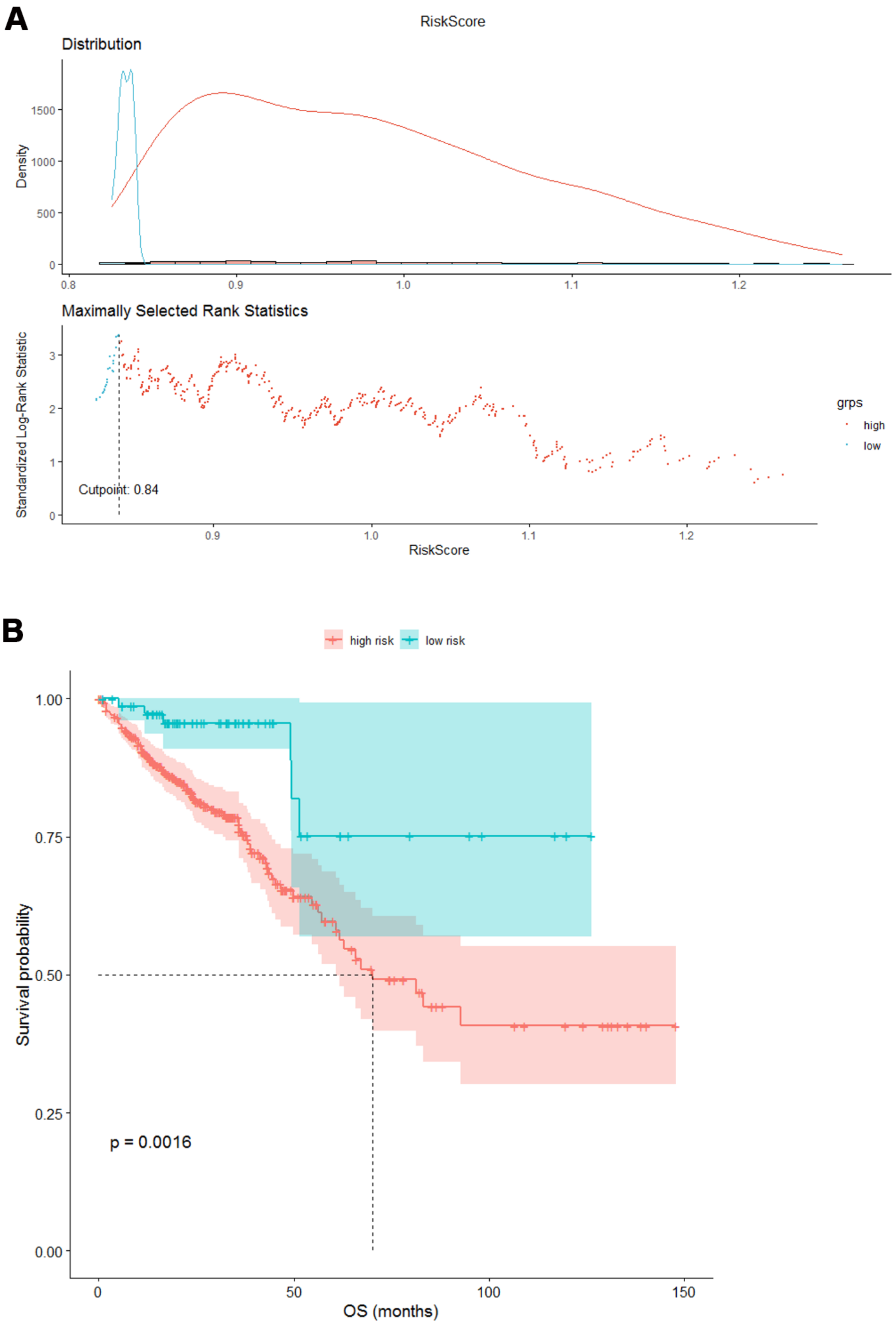

Supplementary Figure 2. (**A**) Upper graph: the distribution of risk scores; lower graph: cutoff point selection based on log rank statistics. (**B**) Kaplan-Meier survival analysis of high risk and low risk groups.

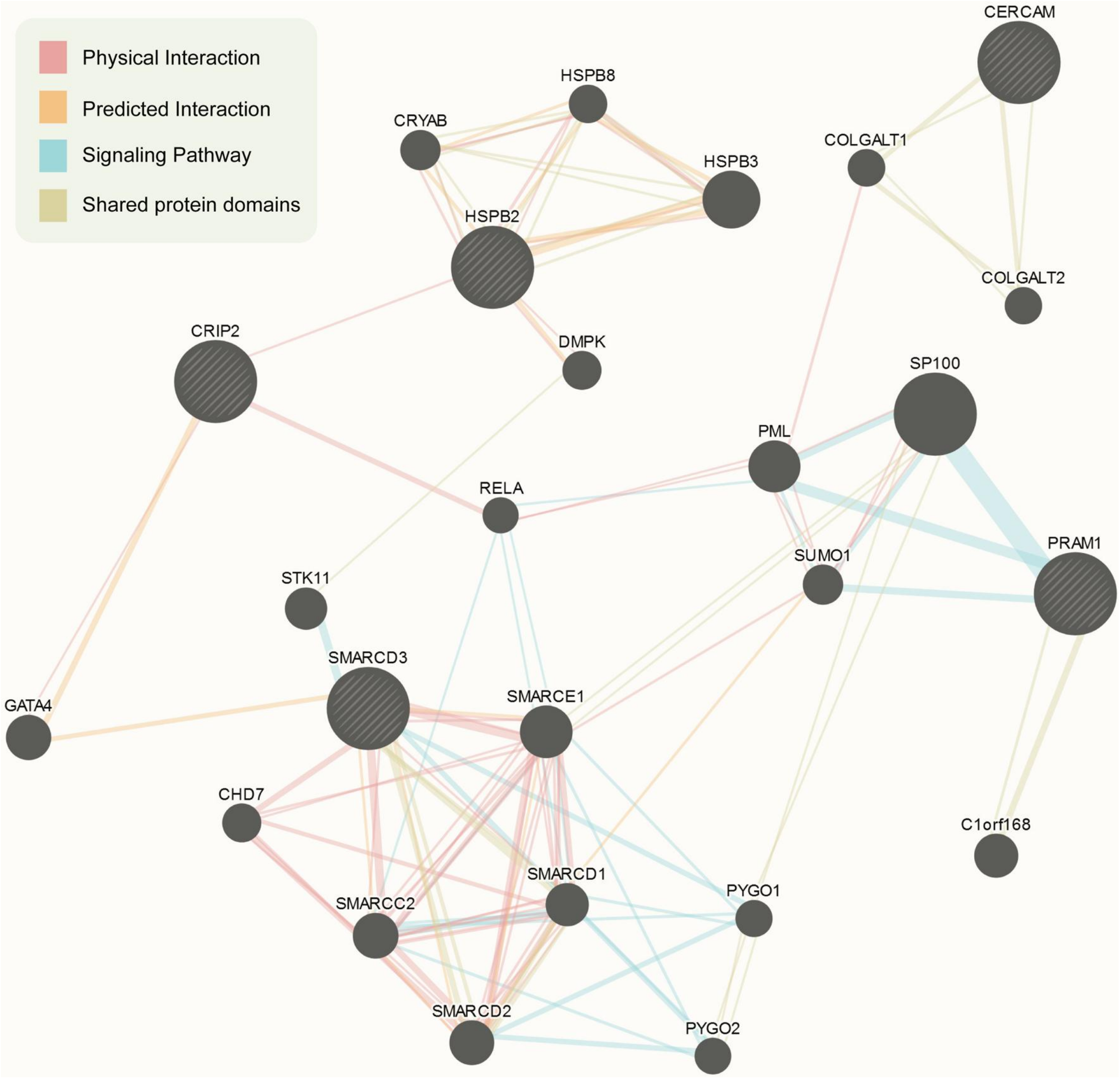

Supplementary Figure 3. Protein-Protein interaction network of SMARCD3, CRIP2, PRAM1, HSPB2 and CERCAM.

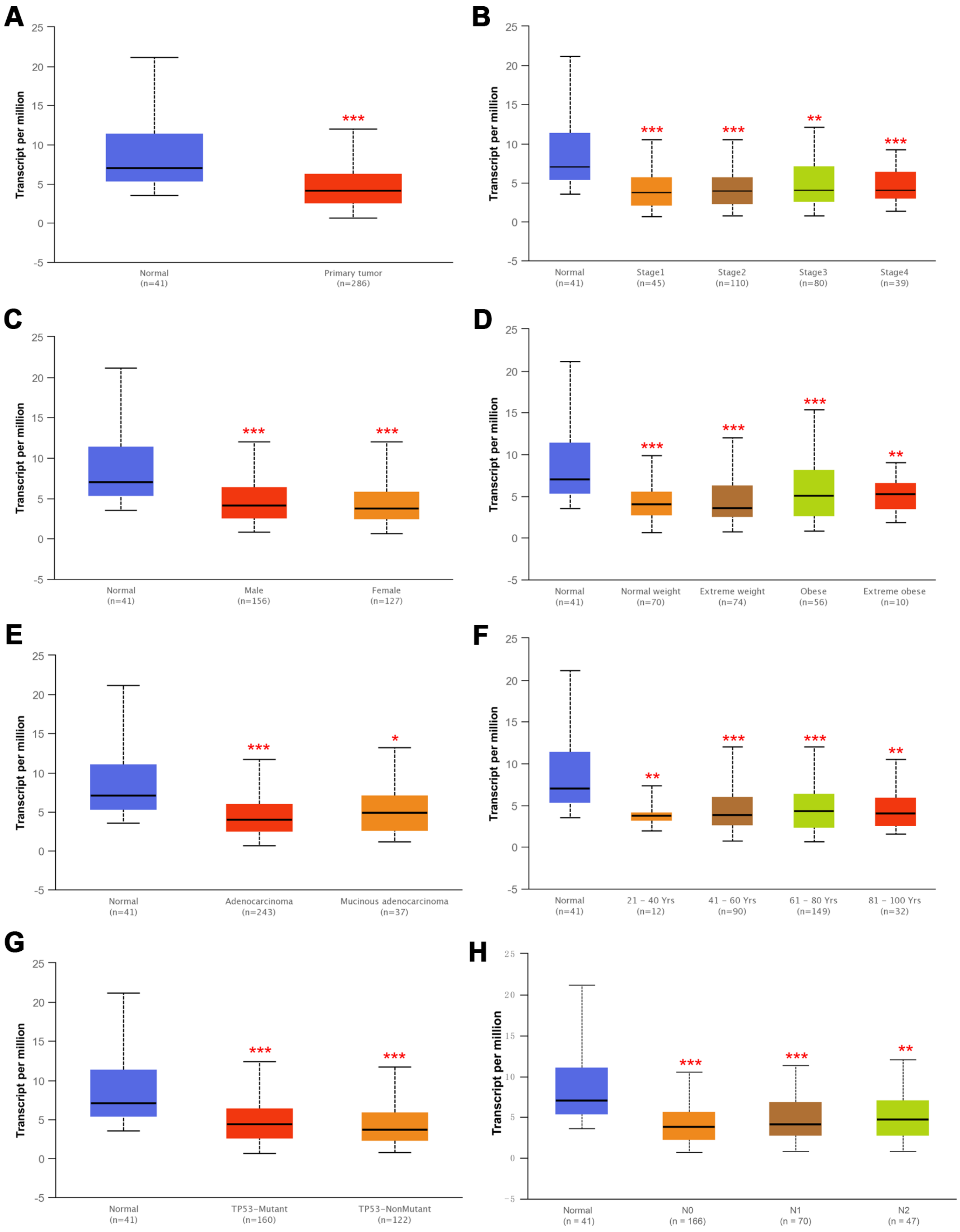

Supplementary Figure 4. SMARCD3 expression in different clinical subgroups. (**A**) SMARCD3 expression in colon cancer and normal controls. SMARCD3 expression in different cancer stage (**B**), gender (**C**), body weight (**D**), sample type (**E**), age (**F**), TP53 mutation status (**G**) and nodal metastasis status (**H**).

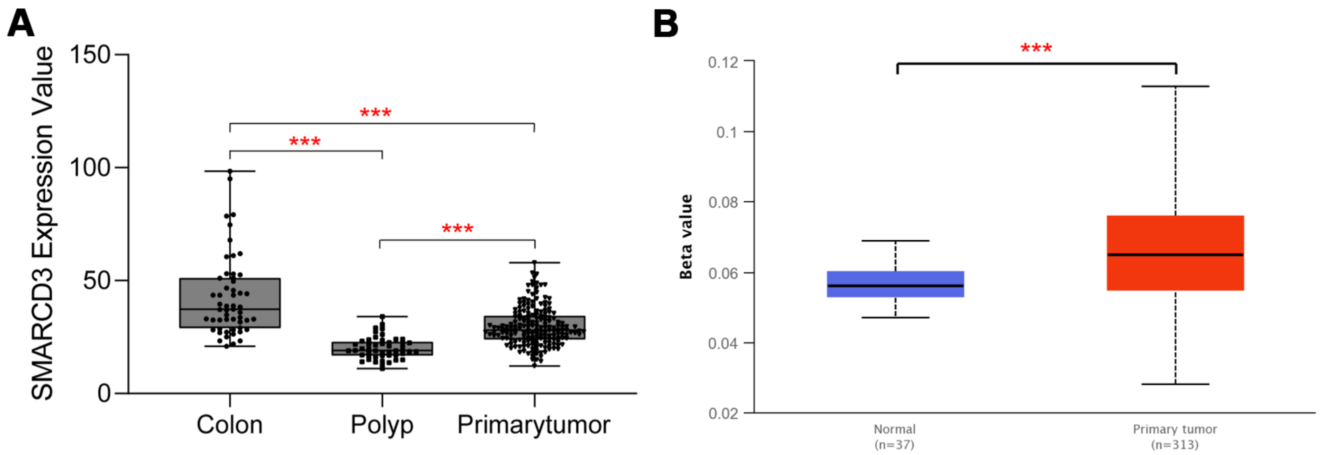

Supplementary Figure 5. (**A**) SMARCD3 expression in normal control, polyps and primary tumor. (**B**) the methylation level of SMARCD3 in primary tumor is significantly higher than normal control.

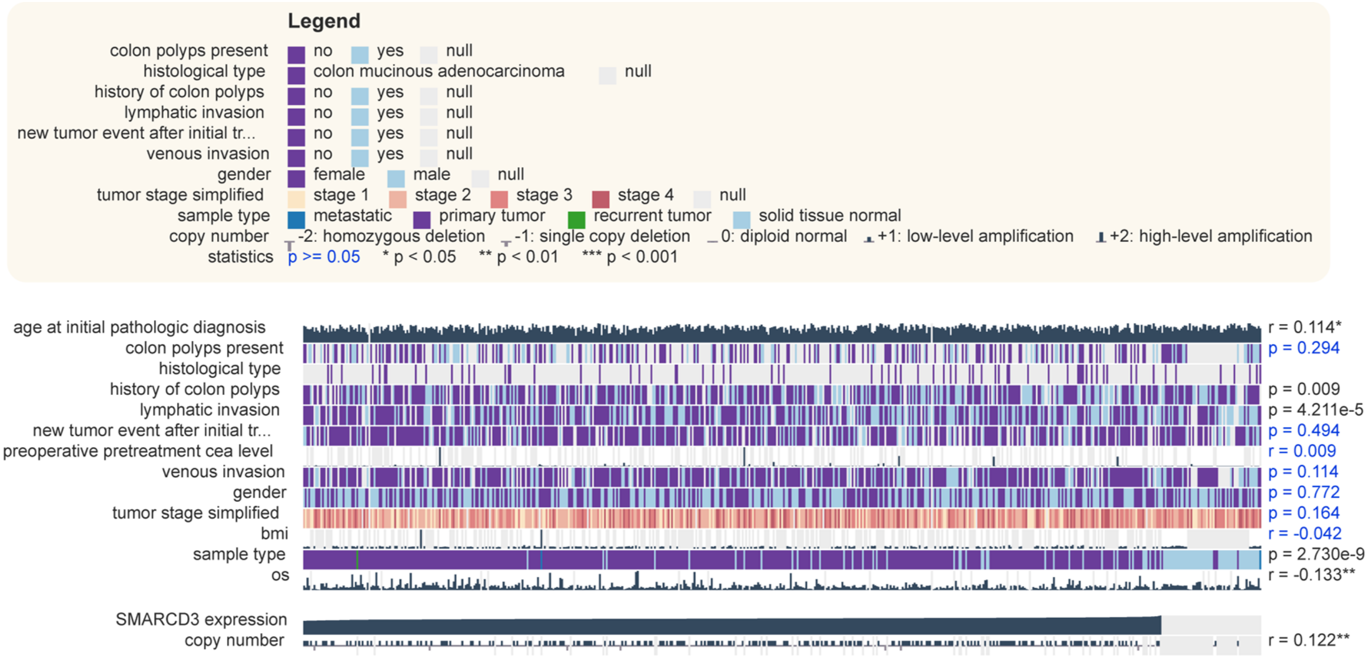

Supplementary Figure 6. Association between SMARCD3 expression levels and clinical features.

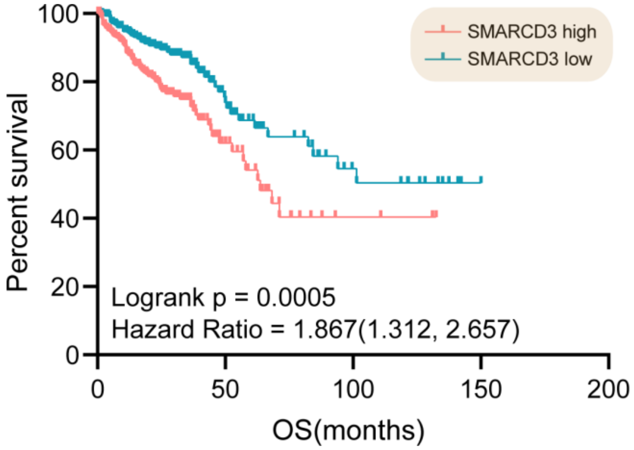

Supplementary Figure 7. Kaplan-Meier survival analysis based on SMARCD3 expression values using TCGA COAD RNAseq data (N = 592).

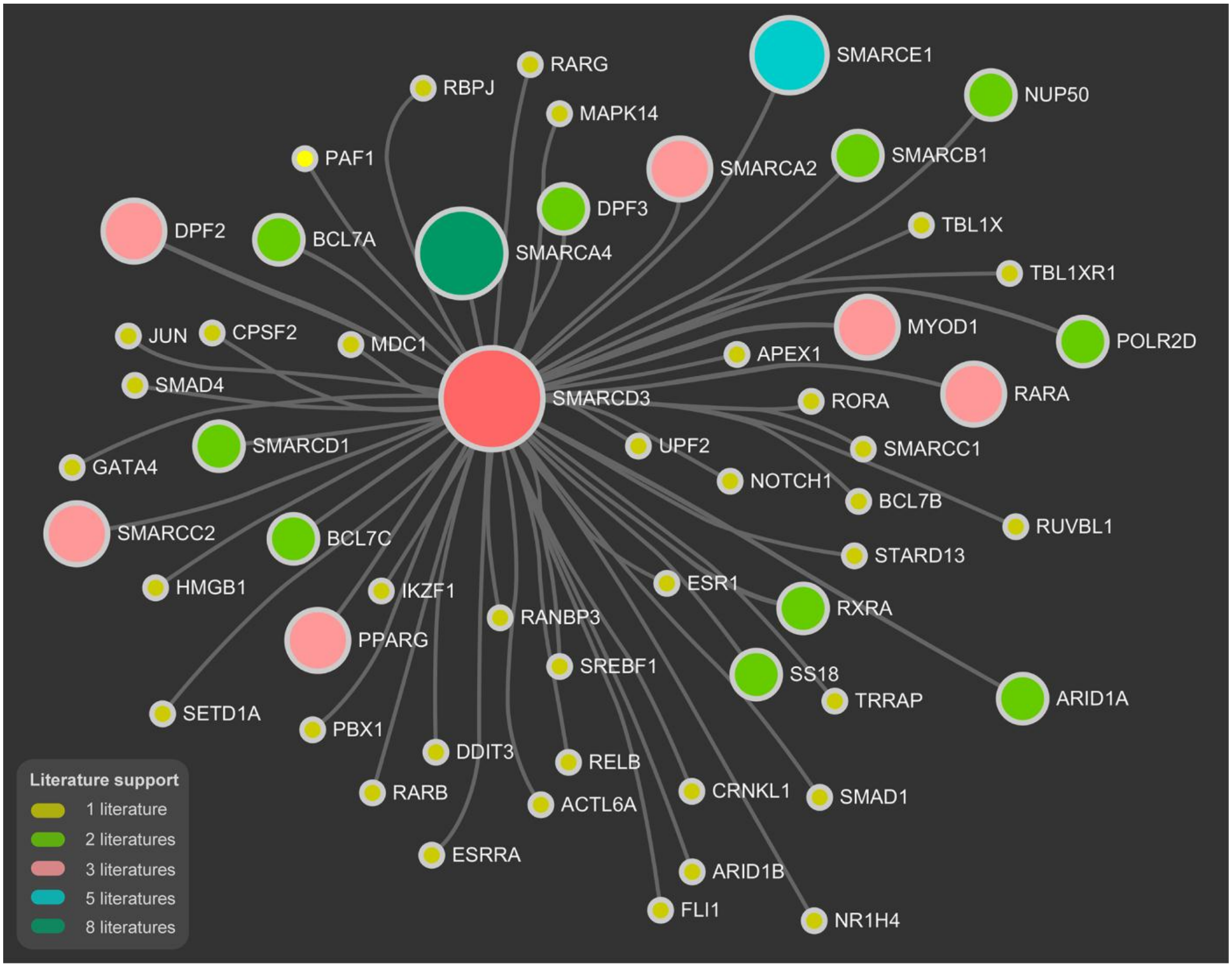

Supplementary Figure 8. Protein-Protein interaction network of SMARCD3 based on data from CTDbase.

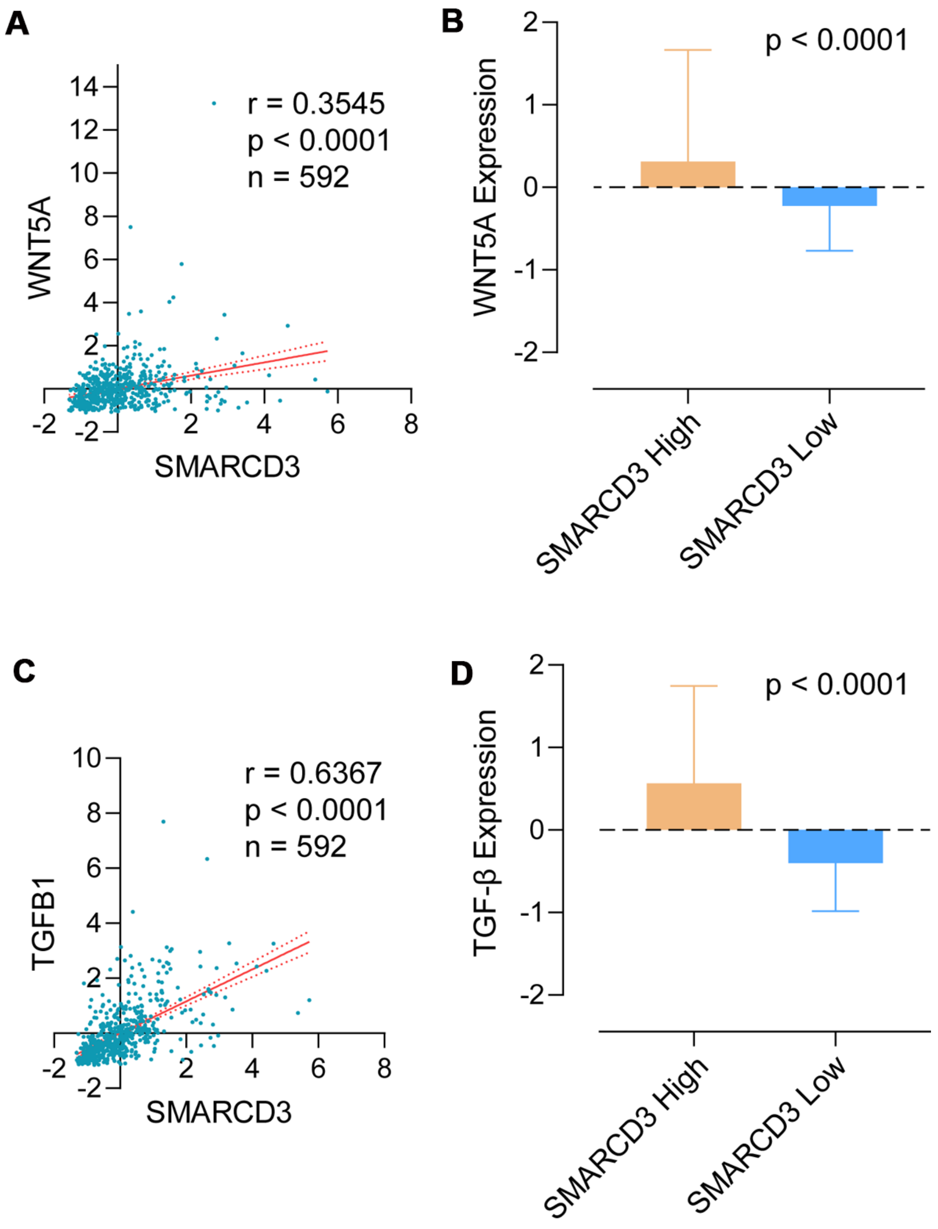

Supplementary Figure 9. SMARCD3 expression is positively correlated with WNT5A and TGF-β. (**A**) Correlation plot of WNT5A and SMARCD3. (**B**) WNT5A mRNA expression in SMARCD3 high and low groups. (**C**) Correlation plot of TGFB1 and SMARCD3. (**D**) TGFB1 mRNA expression in SMARCD3 high and low groups.

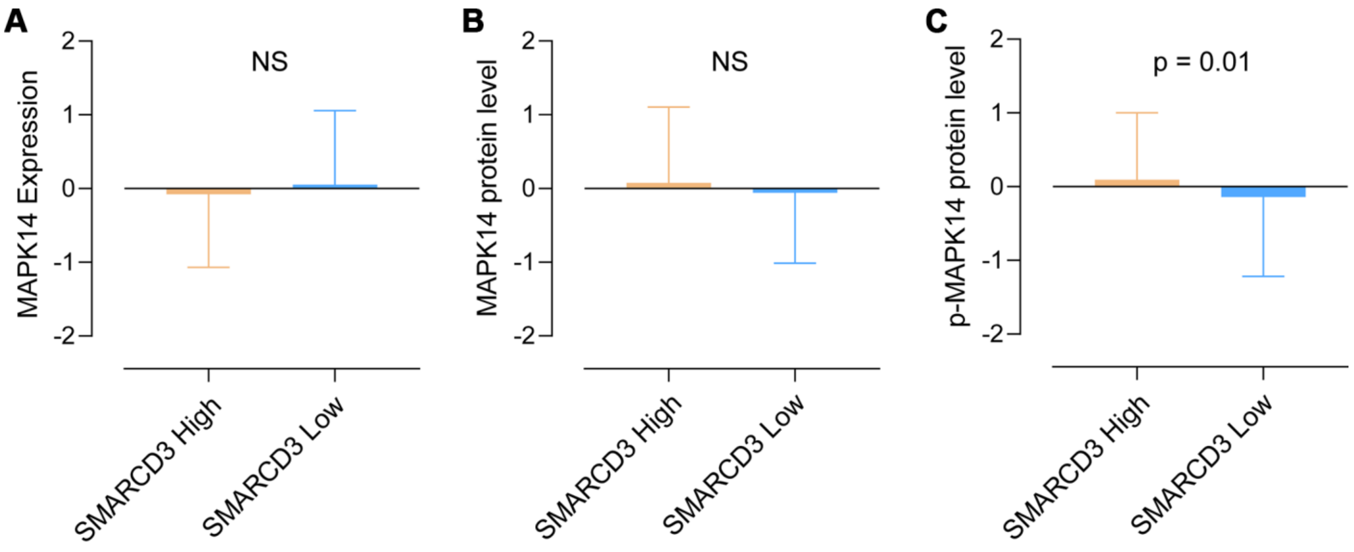

Supplementary Figure 10. SMARCD3 expression is positively correlated with MAPK14 phosphorylation levels. MAPK14 mRNA (**A**), MAPK14 protein (**B**) and p-MAPK14 protein (**C**) expression levels in SMARCD3 high and low groups.
